# Testing the Effectiveness of an Intervention to Improve Romanian Teachers’ LGBT+-Related Attitudes, Cognitions, Behaviors, and Affect: Protocol for a Randomized Controlled Trial

**DOI:** 10.2196/54254

**Published:** 2024-04-23

**Authors:** Ioana Latu, Nastasia Sălăgean, Torill M B Larsen, Andreea Bogdana Isbasoiu, Florin Alin Sava

**Affiliations:** 1 Department of Psychology Faculty of Sociology and Psychology West University of Timisoara Timisoara Romania; 2 School of Psychology Queen's University Beflast Beflast United Kingdom; 3 Department of Scientific Research in Economy, Law and Human-Environment Interaction Institute for Advanced Environmental Research West University of Timișoara Timisoara Romania; 4 Department of Health Promotion and Development Faculty of Psychology at University of Bergen Bergen Norway; 5 Department of Psychology and Education Sciences Transilvania University of Brasov Brasov Romania

**Keywords:** discrimination, intervention, school, lesbian, gay, bisexual, and transgender, attitude, behavior, cognition, stigma, stigmatization, negative impact, physical health, mental health, minority stress model, European, Europe, Romania, stress, young, student, students, undergraduate, bias, data analysis, online intervention, lesbian, gay, bisexual, and transgender stigma, sentiment

## Abstract

**Background:**

Repeated stigmatization due to group membership constitutes a recurrent stressor with negative impact on physical and mental health (minority stress model). Among European countries, Romania ranks low on LGBT+ (lesbian, gay, bisexual, and transgender people. The “+” represents individuals whose identities do not fit typical binary notions of male and female [nonbinary]) inclusion, with 45% of Romanian LGBT+ respondents reporting discrimination in at least 1 area of life in the year preceding the survey. Importantly, while all LGBT+ people might experience minority stress, younger sexual minority individuals are more prone to the detrimental impacts of stigma on their mental and physical health. As such, interventions are necessary to improve the inclusion climate within schools, where young people spend most of their time. Until now, most interventions addressing this topic have been conducted on undergraduate students in Western countries, with no studies conducted in countries that have widespread anti-LGBT+ attitudes.

**Objective:**

This paper describes the research protocol for a randomized controlled trial investigating whether LGBT+ stigma and bias among Romanian school teachers can be reduced using an internet-based intervention focusing on education and contact as primary training elements.

**Methods:**

A sample of 175 school teachers will be randomly assigned to either the control or experimental group. The experimental group participants will receive the intervention first and then complete the outcome measures, whereas the control group will complete the outcome measures first and then receive the intervention. The 1-hour multimedia intervention is developed for internet-based delivery under controlled conditions. It includes 2 interactive exercises, 2 recorded presentations, animations, and testimonies from LGBT+ individuals. Data for attitudinal, behavioral, cognitive, and affective measures will be collected during the same session (before or after the intervention, depending on the condition). We also plan to conduct a brief mixed methods follow-up study at 6 to 8 months post participation to investigate potential long-term effects of training. However, due to attrition and lack of experimental control (all participants will have completed the intervention, regardless of the condition), these data will be analyzed and reported separately using a mixed methods approach.

**Results:**

This paper details the protocol for the teacher intervention study. Data collection began in December 2022 and was completed by February 2023. Data analysis will be performed upon protocol acceptance. Follow-up measures will be completed in 2024. Results are expected to be submitted for publication following analysis in the spring of 2024.

**Conclusions:**

The findings of this study will establish the effectiveness of an internet-based intervention intended to lessen anti-LGBT stigma and sentiment in a nation where these views have long been prevalent. If successful, the intervention could end up serving as a resource for Romanian teachers and guidance counselors in high schools.

**Trial Registration:**

ISRCTN 84290049; https://doi.org/10.1186/ISRCTN84290049

**International Registered Report Identifier (IRRID):**

DERR1-10.2196/54254

## Introduction

### Overview

The experience of stigmatization based on group membership can lead to negative effects on physical and mental health via repeated experiences of stress which, in the long term, accumulate to impact health negatively (minority stress model) [1]. Stigmatization can occur based on membership in any group that is discriminated against. In this research, we focus on LGBT+ (lesbian, gay, bisexual, and transgender people. The “+” symbol represents individuals whose identities do not fit typical binary notions of male and female [nonbinary]) stigmatization and its reduction in Romania.

Among European countries, Romania tends to fare poorly when it comes to LGBT+ inclusion. A recent survey [2] showed that 45% of Romanian LGBT+ respondents felt discriminated against in at least 1 area of life during the year before the survey. Similarly, 43% of respondents experienced harassment due to being LGBT+, a rate that ranks second among all European countries. Much of this discrimination and harassment happens in schools for young people, with 44% of LGBT+ students (15-17 years of age) in Romania saying they are hiding being LGBT+ at school, a rate that is 3 to 4 times higher than in countries such as Luxembourg (11%), the Netherlands (16%), or Malta (17%). Indeed, schools seem to be a fertile ground for LGBT+ harassment, with 51% of incidents involving perpetrators from schools [2], a finding reinforced by a UNESCO (The United Nations Educational, Scientific and Cultural Organization) report on school violence [3].

Such high rates of LGBT+ discrimination among Romanian students are concerning, because stigma impacts young LGBT+ people’s mental and physical health. A meta-analysis [4] including 35 studies of over 2 million heterosexual young people and over 100,000 LGBT+ young individuals (age range 12-20 years) showed that suicide risk was higher for LGBT+ young people compared to those who were not LGBT+. For every heterosexual young person, the chance of suicide was 3.71 higher for gay and lesbian young people, 4.87 higher for bisexual youth, and 5.87 higher for young transgender people. This risk is paired with a higher likelihood for sexual minority youth to experience mental health issues such as anxiety and depression but also impaired academic performance [5]. The findings for sexual minority youth are consistent with sexual minority individuals in general—a systematic review showed that the majority of studies revealed a higher risk for mental health issues, substance abuse, and suicide risk for sexual minorities [6]. LGBT+ people are also more likely to endure physical health issues due to minority stress, including an increased risk for cancer, cardiovascular disease, and chronic conditions such as asthma and diabetes [7].

Importantly, although minority stress can affect all LGBT+ people, younger sexual minority individuals seem to be most vulnerable to the negative effects of stigma on their mental and physical health. A meta-analysis indicated age as a significant moderator, such that youth younger than 17 years of age experienced higher negative outcomes due to LGBT-related victimization compared to those older than 17 years of age [8].

Together, these findings indicate a strong need to implement effective interventions to reduce LGBT+ stigma and bias in Romanian schools, to improve the mental and physical health of LGBT+ students in Romania. These interventions may be most efficient particularly among teachers, as they can impact their students’ experiences either directly or indirectly, via establishing inclusivity norms in the classroom.

To be effective, interventions should not only focus on reducing teachers’ biases toward LGBT+ students, but also on learning, implementing, and modeling behaviors that would equip them to intervene when students experience LGBT+-related victimization. A meta-analysis [9] showed that interventions can be moderately successful in reducing sexual prejudice, resulting in changes ranging from one-third to one-half of an SD in size. Moreover, the meta-analysis specified the most effective strategies in reducing different outcomes—educational interventions, contact with LGBT+ individuals, and interventions combining education with contact. A more careful analysis reveals that most intervention studies have been conducted on undergraduate students in Western countries and none in Eastern Europe or other countries with strong antigay attitudes. Although more recent research has started to test interventions in other countries such as Jamaica [10] or Brazil [11], no such adaptation exists in Romania (or Eastern Europe), particularly for teachers. Adapting interventions to the cultural and institutional context seems vital, given that a recent qualitative analysis suggests that participants tend to criticize many interventions for their mismatch with the context in which they are conducted, possibly as a rationale for resisting change [12].

The primary goal of this research line is to design and test an intervention that is geared toward Romanian teachers. Importantly, we plan to achieve this goal by deliberately taking into account specific cultural and institutional characteristics, rather than indiscriminately applying previous intervention strategies. More specifically, although we plan to use education and contact as primary training elements, consistent with meta-analytic findings [9], we plan to design the educational components to respond to the needs of our particular target group. For example, we drew from recent research conducted in Russia [13], a country with a similar culture and history regarding LGBT+ attitudes. Building on these findings, we propose that the educational component should include information regarding the biological (vs social) causes of homosexuality, given that attributions of causality are related to perceived threat, and subsequently lead to biased outcomes. In addition, given the importance of threat in predicting antigay bias, and consistent with intergroup threat theory [14], education should explicitly address potential feelings or perceptions of threat (for example, the perception that exposure to LGBT+ people will “make” children gay).

A second novel aspect is that the intervention will aim not only to improve attitudes and knowledge as an outcome but also target behavioral change by imparting tools that teachers can use to address LGBT+-related victimization in schools. This strategy has been used successfully in the past in a web-based intervention program for Brazilian health practitioners [11]. As a result, we are likely to increase the impact of the intervention of LGBT+ students, not only by improving the inclusion climate within the schools but also by improving teachers’ actual behavioral intentions and skills that may improve LGBT+ students’ outcomes.

### Aims and Hypotheses

In this research, we design and report the protocol of a randomized controlled trial (RCT) intervention and evaluation. The intervention is based on existing evidence about the efficacy of strategies identified in Bartoş et al’s [9] meta-analysis. According to their results, the most effective strategies in reducing LGBT+ stigma combine educational elements with contact with LGBT+ people. We also add elements that were found to be useful in other bias-reducing interventions such as perspective taking [15] and self-efficacy [16,17], and which we deem culturally appropriate. A summary of intervention components, subcomponents, and contents is presented in Table 1. For each element, we include the supporting reference, as an evidence base for their efficiency in reducing bias.

Using an RCT in which teachers are randomly assigned to complete the intervention before assessing outcomes (experimental) or after assessing outcomes (control), we predict that the intervention will have a positive effect on attitudinal, cognitive, behavioral, and affective measures of LGBT+ bias among teachers. Specifically, we predict that teachers randomly assigned to the experimental condition, compared to those randomly assigned to the control condition will experience (1) more positive attitudes toward LGBT+ individuals, (2) more factual knowledge about LGBT+ issues, (3) stronger behavioral intentions and self-efficacy about addressing LGBT+ issues in the classroom, and (4) more positive affect toward LGBT+ individuals.

**Table 1:** Training elements for the randomized controlled trial intervention designed to improve Romanian teachers’ LGBT+^a^ related attitudes, cognitions, behaviors, and affect^b^.

Intervention component and subcomponent		Duration and timestamps	Content and supporting references
Introduction		Duration: 02 minutes 50 seconds Timestamps: 00.00-02.50	Introduction from academic research team, highlighting the goals and the evidence-based nature of the intervention
**Education** **[9]**			
	Definition of terms	Duration: 05 minutes 40 seconds Timestamps: 02.50-08.30	Definition of LGBT+ terms Definition of sexual orientation Differentiation between sex versus gender Definition of intersex Description of gender identity and transgender
	Threat reduction	Duration: 03 minutes 40 seconds Timestamps: 08.30-12.10	LGBT+ children and their families: meta-analysis on children raised by LGBT+ families Sexuality and sexual orientation in humans and animals. Normalizing varied sexual orientations across species The function of same-sex sexual behavior in ensuring survival
	Effects of stigma to understand minority stress [1]	Duration: 05 minutes 35 seconds Timestamps: 12.10-17.45	Suicide rates of LGBT+ youth The role of stress from exposure to prejudice, ridicule, physical, and verbal aggression in understanding mental health outcomes The role of social support and family or school acceptance in improving mental health and the danger of conversion therapy Bullying as a special case of discrimination, types of bullying, the role of sexual orientation
	Behavioral tools [11]	Duration: 04 minutes 00 second Timestamps: 24.45-28.45	Presentation of tools that can be used by teachers, including the following: Romania’s new antibullying law and bullying protocol in schools Lesson plans to discuss and combat bullying in the classroom UNICEF^c^ intervention model for bullying Procedure to report bullying and harassment with the Romanian National Council for Combating Discrimination Support groups for LGBT+ students in Romania For all resources links were shared in the presentation and then sent in PDF format to all participants
**Perspective taking [15]**			
	Writing exercise, 7 minutes	Duration: 07 minutes 00 second Timestamps: 17.45-24.45	Writing prompt: “Imagine a day in the life of a gay student—write a few paragraphs about what this student is living and feeling on a school day. What are his/her thoughts and feelings that day? Please write down your answers.”
**Contact [9,10]**			
	Indirect contact via recorded testimonials	Duration: 15 minutes 00 second Timestamps: 28.45-43:45	Video testimonials of 3 university students (1 gay man, 1 bisexual woman, and a lesbian woman) and 1 teacher (lesbian woman). Testimonial prompts for students included the following: Tell us about yourself When did you come out? What were your barriers during this process How was your experience in school? Were there teachers who supported you? What would you have needed when you were a high school student? What would you say to teachers to help them support their LGBT+ students? Testimonial prompts for the teacher included the following: Tell us about yourself How do you see LGBT+ students’ experiences in schools currently? What is missing from our educational system in supporting LGBT+ students? What would you say to teachers to help them support their LGBT+ students?
**Self-efficacy**			
	Writing exercise, 5 minutes	Duration: 5 minutes 00 second Timestamps: 43.45-48:45	Writing prompt: “Imagine now that, as a teacher, you are interacting with the student you previously imagined. For example, the student asks for your help in a discussion after class. Knowing what you know now about the LGBT+ community and how we can help LGBT+ students, please imagine this interaction in which you offer suggestions and support. What would you say to this student? Please write down your answers.”
Conclusion		Duration: 1 minutes 10 seconds Timestamps: 48.45-49:55	Acknowledging the advisory board representatives from the LGBT+ community for their input and feedback

^a^LGBT+: lesbian, gay, bisexual, and transgender people. The “+” symbol represents individuals whose identities do not fit typical binary notions of male and female (nonbinary).

^b^All intervention elements are drawn from empirical evidence. We present timestamps and specific content which is included in the multimedia intervention resource.

^c^UNICEF: United Nations Children's Fund.

## Methods

### Participants and Recruitment

We plan to collect data exclusively from teachers or counselors working in Romanian public schools. All participants will be required to speak Romanian because of the language used in the intervention and measures. Participants who complete the intervention will be sent a participation certificate which could be used for continuing education credits and are given the option to enter a raffle where they can win a 500 RON (equivalent to US $110) gift certificate.

Our recruitment strategy is to ask the local Center for Educational Resources and Assistance to distribute a message nationally, to all their members who are teachers or counselors employed in Romanian schools. If interested, they have the opportunity to sign up for 1 of the several sessions depending on their availability. The sessions are capped at 30, with an average of 25 participants signing up for each (range 24-27). We will advertise a total of 17 sessions across 3 months at different times during the day.

### Study Design

This intervention’s experimental design is an RCT. We use a 2-group random assignment design, such that participants are randomly assigned to either the experimental condition in which they receive the intervention first and then complete the outcome measures, or to a control condition in which they complete the outcome measures first and then receive the intervention. From an ethical perspective, we chose this design so that all participants will receive the intervention and associated resources by the end of their participation. From a validity perspective, the outcome measures of those in the control condition are not affected by the intervention, thus serving as an appropriate control comparison to participants in the experimental condition.

Participants will join sessions in groups of up to 30 participants, depending on their availability. Cluster randomization will be done at the session level, such that each session will be randomly assigned to either experimental or control conditions. Individual randomization within the session is not possible given that the intervention will be presented to all participants at the same time. Sessions will be scheduled across different times and days outside typical working hours, so we do not anticipate any systematic biases arising from participants’ session choices. To ensure experimental control, each session will be led by 2 researchers who deliver scripted instructions, answer questions, and ensure all participants complete the study at a similar pace and with minimal distraction.

### Intervention

The intervention is designed for internet-based delivery and is multimedia, containing a recorded presentation, animations, testimonials of LGBT+ people, as well as 2 exercises. Whereas the intervention is rooted in empirical evidence, we also ensured cultural sensitivity by collaborating with local LGBT+ educational and advocacy nongovernmental organizations who offered feedback on intervention components. A detailed summary of the evidence-based components, as well as timestamps and duration for each subcomponent, are included in Table 1.

### Ethical Considerations

Ethics approval was obtained from the West University of Timisoara, Romania, ethical panel (74505/10.11.2022), based on an application containing the procedure, measures, and materials used. Upon being presented with detailed information regarding the study, informed consent will be obtained from all participants before starting their participation. All data will be anonymously collected, with no personal or identifiable information being recorded. All fully anonymous data will be stored on password-protected computers and servers. Compensation consists of a participation certificate which could be used for continuing education credits and the option to enter a raffle where they can win a 500 RON (equivalent to US $110) gift certificate. There is no identification of individual participants in any images within the paper.

### Outcomes

#### Overview

With 1 exception (the factual knowledge variable), we will use validated scales to measure intervention outcomes. Where possible, we will use previously translated and validated scales in Romanian, selected from ResearchCentral repository, a free internet-based platform dedicated to the development of Romanian psychology by providing researchers with free, validated assessment tools. Where translations did not previously exist, 2 Romanian researchers, who are fluent in English, translated and then back-translated the scales to ensure accuracy. For all scales, before computing a final score, we will ensure sufficient reliability by computing Cronbach α, with a cutoff minimal score of 0.70.

#### Attitudinal

##### Overview

Given the wide divergence in the definition of antigay bias or homophobia, several scales have been developed across time [18] and will be used in this research.

##### Attitudes Toward Lesbians and Gay Men Scale

This 10-item scale measures beliefs and attitudes toward gay men and lesbians (“Sex between two men is just plain wrong” and “Female homosexuality is a perversion”) [19]. Items are rated on a Likert scale from 1 (strongly disagree) to 5 (strongly agree). After reverse coding 4 items, scores will be averaged into a final score with higher values denoting more negative attitudes.

##### Homophobia Scale

This 25-item scale measures attitudes but also social avoidance and aggression toward gay people (“If I discovered a friend was gay I would end the friendship” and “I tease and make jokes about gay people”) [20]. Items are rated from 1 (strongly disagree) to 5 (strongly agree) on a Likert scale. After reverse coding 9 items, scores will be averaged into a final score with higher values denoting more negative attitudes.

##### Attitudes Toward Homosexuals Scale

This 12-item scale measures attitudes toward gay people (“Homosexuality is disgusting in the eyes of God” and “If I can, I prefer to not be in the company of homosexuals”) [21]. Items are rated from 1 (strongly disagree) to 5 (strongly agree) Likert scale. After reverse coding 5 items, scores will be averaged into a final score with higher values denoting more negative attitudes.

#### Behavioral

##### Behavioral Intentions

This 16-item scale measures intentions for supportive professional behaviors that teachers would perform in the classroom (“I would talk with a student about questions regarding sexual orientation” and “I would have books about gay and lesbian issues in my classroom”) [22,23]. Items are rated on a Likert scale from 1 (strongly disagree) to 5 (strongly agree). Scores will be averaged into a final score with higher values denoting more willingness to engage in LGBT+ supportive behaviors in the classroom.

##### Self-Efficacy

This 10-item scale adapted the original scale to working with LGBT+ students in the school context (“If I try hard, I can solve difficult issues related to LGBT+ students” and “I can deal with unexpected situations that arise with LGBT+ students”) [24]. Items were rated from 1 (Not at all true for me) to 4 (Perfectly true for me). Scores will be averaged into a final score with higher values denoting more self-efficacy in dealing with LGBT+-related behaviors in the classroom.

#### Cognitive: Factual Knowledge About LGBT+ Issues

We constructed 7 items based on training content to assess participants’ knowledge about LGBT+ issues (“Gender is a biological construct, unrelated to cultural associations” and “Heterosexual youth are 1.5 to 3 times more likely to attempt suicide compared to LGBT+ youth”). Items are rated as true or false, and a final score will be computed by adding up the number of correct responses, with higher scores denoting more knowledge about LGBT+ issues.

#### Affective

##### Feeling Thermometer (1 Item Each for Gay, Lesbian, and Bisexual)

We will use a feeling thermometer [25] to assess participants’ feelings toward gay, lesbian, and bisexual people by asking them to rate how they feel about each group using a slider thermometer scale from 0 (very negative feeling) to 100 (very positive feeling).

##### Perspective Taking

This 5-item measure assesses the extent to which participants take the perspective of LGBT+ people (“Can you imagine how an LGBT+ person feels?” and “Do you have an understanding of issues that are important for LGBT+ people”) on a scale from 1 (Never) to 5 (All the time) [26]. Scores will be computed by averaging the 5 items, with higher final scores denoting more perspective-taking.

##### Intergroup Disgust Sensitivity

We adapted this 7-item scale to measure repulsion toward LGBT+ groups (“I feel disgusted when people with a different sexual orientation invade my personal space” and “After shaking hands with someone who has a different sexual orientation, even if their hands were clean, I would want to wash my hands.”) [27]. Items are rated from 1 (strongly disagree) to 5 (strongly agree) on a Likert scale. After reverse scoring 1 item, scores will be averaged into a final score with higher values denoting more disgust toward LGBT+ outgroups.

##### Intergroup Anxiety

This 10-item scale measures anxiety-related emotions when interacting with people of another sexual orientation by asking them to rate their likelihood of feeling several emotions (embarrassed, unsure, irritated, suspicious, etc) on a scale from 1 (not at all) to 10 (extremely) [28]. A total of 2 scores will be computed, 1 for positive emotions and another for negative emotions.

##### Toronto Empathy Questionnaire

This 16-item scale measures the general tendency to empathize with other people (“It upsets me to see someone being treated disrespectfully” and “I find that I am ‘in tune’ with other people’s moods”) [29,30]. Items are rated on a Likert scale from 1 (strongly disagree) to 5 (strongly agree). After reverse scoring 7 items, scores will be averaged into a final score with higher values denoting more empathy.

#### Demographic and Control Variables

##### Demographics

It consists of age, gender, and sexual orientation.

##### Contact With LGBT+ People

We will ask participants how often they have contact (eg, talk) with a gay man, a lesbian woman, a bisexual woman or man, or a transgender person on a scale from 1 (almost daily) to 6 (never).

##### Religiosity

Religiosity (Duke University Religion Index) [31] is a 5-item scale measure of religious involvement, measuring organizational religious activity, nonorganizational religious activity, and intrinsic religiosity (or subjective religiosity). Responses are averaged in 1 single score with higher scores denoting more religiosity.

##### Ideology

Ideology was measured by asking people to indicate their political orientation on a 100-point sliding scale from 0 (very conservative) to 50 (center) to 100 (very liberal or progressive).

### Statistical Analysis

#### Power

To determine our sample size, we conducted a power analysis using an average effect size of *d*=0.66 computed from Bartoş et al’s [9] meta-analysis to ensure a statistical power of 0.80. The analysis indicated a sample of 122, but we aim to overrecruit, if possible, up to 200 participants given multiple outcomes. We do not have a stopping rule—we will recruit until all interested participants are given the chance to participate within 1 of the 17 sessions posted.

#### Data Exclusion

We will use data from all participants who completed their participation in the study, without any exclusions. Before computing final scores for each outcome variable, we will ensure sufficient reliability using Cronbach α, using a threshold of 0.70. To test our hypotheses, we will perform a series of 1-way between-subjects ANOVAs to compare attitudinal, cognitive, behavioral, and affective outcomes between the participants in the experimental and control conditions. For the effective measures we will perform the analyses (1) while controlling for contact with LBGT+ individuals and (2) separately by LGBT+ status (given the nature of the measures). We will report *F* test and *P* values, as well as all descriptive statistics (n, mean, and SD). We will also compute Cohen *d* for estimating the effect size, using means and SDs. Violin plots in R ggplot2 will also be included to visually represent means, CIs, as well as score distributions for each outcome.

## Results

This paper details the protocol for the teacher intervention study. Data collection began in December 2022 and was completed by February 2023. Data analysis will be performed upon protocol acceptance. Follow-up measures will be completed in 2024. Results are expected to be submitted for publication following analysis in the spring of 2024.

## Discussion

This paper describes the research protocol design and planned evaluation of an RCT aimed at improving attitudinal, cognitive, behavioral, and affective outcomes in Romanian teachers regarding LGBT+ inclusion. Strengths of the intervention include its evidence-based nature—all components included were derived from research showing positive outcomes on LGBT+ or other bias reduction. All elements were, however, adapted to be appropriate and sensitive to the Romanian cultural system and educational context. The intervention was also engaging and multimedia, thus increasing engagement. Importantly, it offers evidence-based tools to further address LGBT+ biases and bullying in schools, thus, potentially leading to stronger behavioral effects.

Limitations include the fact that outcomes will be assessed immediately after the intervention, so we are unsure about the long-term effects, as well as whether potential positive effects further impact LGBT+ students’ lives. Further studies should investigate the effects on a larger sample of teachers, with a wider range of initial attitudes toward LGBT+ inclusion.

If successful, however, the intervention has the potential of becoming a valuable, nationally available resource for teachers and high school counselors across Romania. The materials developed, especially those around education, perspective-taking, and self-efficacy can be further used to educate students about LGBT+ issues and increase their willingness and capacity to support LGBT+ peers. The findings resulting from this protocol are important, as they are the first to test the effectiveness of an evidence-based intervention in improving LGBT+ stigma in Romanian schools.

## Abbreviations

LGBT+: lesbian, gay, bisexual, and transgender people. The “+” symbol represents individuals whose identities do not fit typical binary notions of male and female (nonbinary)
RCT: randomized controlled trial
UNESCO: The United Nations Educational, Scientific and Cultural Organization

## Data Availability

The data sets generated and analyzed during this study will be published in anonymous form on Open Science Framework and the European Commission Funded Research (OpenAire) community platform. This will be done after the successful publication of the results.
